# High‐intensity interval training improves cardiomyocyte contractile function and myofilament sensitivity to intracellular Ca^2+^ in obese rats

**DOI:** 10.1113/EP092015

**Published:** 2024-08-29

**Authors:** Matheus Corteletti dos Santos, Daniel Sesana da Silva, Jóctan Pimentel Cordeiro, Lucas Furtado Domingos, Ezio Henrique da Silva Gomes, Breno Valentim Nogueira, Danilo Sales Bocalini, Ana Paula Lima Leopoldo, André Soares Leopoldo

**Affiliations:** ^1^ Postgraduate Program in Physiological Sciences, Health Sciences Center Federal University of Espírito Santo Espírito Santo Vitória Brazil; ^2^ Postgraduate Program in Physical Education, Center of Physical Education and Sports Federal University of Espírito Santo Espírito Santo Vitória Brazil; ^3^ Postgraduate Program in Nutrition and Health, Health Sciences Center Federal University of Espírito Santo Espírito Santo Vitória Brazil; ^4^ Postgraduate Program in Biotechnology, Health Sciences Center Federal University of Espírito Santo Espírito Santo Vitória Brazil; ^5^ Department of Morphology, Health Sciences Center Federal University of Espírito Santo Espírito Santo Vitória Brazil; ^6^ Department of Sports, Center of Physical Education and Sports Federal University of Espírito Santo Espírito Santo Vitória Brazil

**Keywords:** calcium handling, cardiac remodelling, high‐intensity interval training, obesity

## Abstract

High‐intensity interval training (HIIT) has shown significant results in addressing adiposity and risk factors associated with obesity. However, there are no studies that investigate the effects of HIIT on contractility and intracellular Ca^2+^ handling. The purpose of this study was to explore the impact of HIIT on cardiomyocyte contractile function and intracellular Ca^2+^ handling in rats in which obesity was induced by a saturated high‐fat diet (HFD). Male Wistar rats were initially randomized into a standard diet and a HFD group. The experimental protocol spanned 23 weeks, comprising the induction and maintenance of obesity (15 weeks) followed by HIIT treatment (8 weeks). Performance was assessed using the maximum oxygen consumption test (${\dot{V}}_{O_{2}\max}$). Evaluation encompassed cardiac, adipose and skeletal muscle histology, as well as contractility and intracellular Ca^2+^ handling. HIIT resulted in a reduction in visceral area, an increase in ${\dot{V}}_{O_{2}\max}$, and an augmentation of gastrocnemius fibre diameter in obese subjects. Additionally, HIIT led to a decrease in collagen fraction, an increase in percentage shortening, and a reduction in systolic Ca^2+^/percentage shortening and systolic Ca^2+^/maximum shortening rates. HIIT induces physiological cardiac remodelling, enhancing the contractile function of cardiomyocytes and improving myofilament sensitivity to Ca^2+^ in the context of obesity. This approach not only enhances cardiorespiratory and physical performance but also reduces visceral area and prevents interstitial fibrosis.

## Abstract (highlights)

- **What is the central question of this study?**
  Does high‐intensity interval training (HIIT) enhance myocardial contractility in models of obesity induced by a saturated high‐fat diet, and does it promote an improvement in intracellular Ca^2+^ handling (Ca^2+^ influx and reuptake)?
- **What is the main finding and its importance?**
  HIIT, as a non‐pharmacological tool of short duration but high intensity, improves cardiovascular function and the sensitivity of myofilaments to intracellular Ca^2+^ handling, as well as promoting a reduction in visceral fat area. Thus, the obesity‐induced impairment of the heart's contractile properties was partially prevented by an 8‐week HIIT programme.

## 1 INTRODUCTION

Obesity is a chronic inflammatory disease characterized by excessive accumulation of body fat, leading to a decrease in quality of life and life expectancy (Abel et al., 2008). Lifestyle changes, such as a diet rich in calorically dense foods, increased consumption of simple sugars and fats, low intake of complex carbohydrates, and reduced caloric expenditure due to a sedentary lifestyle and/or physical inactivity, contribute to elevated adiposity, resulting in overweight and obesity (Abel et al., 2008; Van Hul & Cani, 2023).

Data from a systematic bibliographic review investigating cardiac remodelling in obesity (Abel et al., 2008) indicate an association between obesity and myocardial contractility dysfunction. Findings suggest that obesity is linked to left ventricular (LV) interstitial fibrosis, leading to pathological LV hypertrophy, impairing systolic function, and affecting functional parameters such as shortening, Ca^2+^ sensitivity of myofilaments and Ca^2+^ handling (Abel et al., 2008). Leopoldo and collaborators (2011) observed compromised myocardial responsiveness to a post‐rest contraction stimulus and increased extracellular Ca^2+^ in the isolated papillary muscle from obese rats induced by a high‐fat diet (HFD) for 15 weeks. Ren (2007), using obese mice induced by a HFD for 16 weeks, showed prolonged time‐to‐90% lengthening, depressed peak shortening (PS) and maximal velocity of shortening/lengthening without affecting intracellular Ca^2+^ properties. Additionally, Hun and Zhang (2017) found that changes in contractility parameters occur even after 12 weeks of inducing obesity with a HFD, demonstrating that dysfunctions begin early in obesity.

Studies aimed at understanding the mechanisms of contractility dysfunction and Ca^2+^ handling have demonstrated that cardiac dysfunction resulting from obesity may be due to damage to intracellular Ca^2+^ handling (Coelho et al., 2023; Guatimosim et al., 2001; Leopoldo et al., 2011). As demonstrated previously, studies have indicated Ca^2+^ handling damage in obesity (Abdurrachim et al., 2014; Guatimosim et al., 2001; Leopoldo et al., 2011).

Various treatment strategies for obesity are reported in the literature, including changes in eating patterns, medications, behavioural changes and physical exercise (Yu et al., 2023). Although the exercise used in the treatment of obesity is predominantly aerobic exercise, practiced continuously and at a constant intensity, some studies have demonstrated that high‐intensity interval training (HIIT) also presents important results in relation to body adiposity reduction and the reversal of risk factors associated with obesity (Ferretti et al., 2022; Lee et al., 2024; Sene‐Fiorese et al., 2008; Yu et al., 2023).

HIIT is an exercise approach that stands out for the alternation between periods of high and low intensity, accompanied by active or passive recovery. This methodology allows for the execution of vigorous exercises with a higher training volume and intense work intervals ranging from 5 s to 8 min, performed at an intensity between 85% and 95% of the estimated maximum heart rate. Recovery phases, in turn, occur at an intensity of 40–50% of the estimated maximum heart rate (Chang et al., 2021; Taylor et al., 2020; Yu et al., 2023).

Compared to continuous aerobic exercise, HIIT differs due to its short duration and the alternation between extreme intensities, while continuous aerobic exercise typically has a longer duration (40 min or more) with moderate intensity. This distinction highlights the unique ability of HIIT to promote rapid and effective adaptations in cardiovascular and metabolic systems (Bo et al., 2023; Yu et al., 2023). In recent years, HIIT has become an important non‐pharmacological tool in promoting health. Compared to classic aerobic training, it has demonstrated similar or better effects on visceral and liver fat loss, improving insulin resistance (IR) and cardiovascular function while being more time‐efficient (Bo et al., 2023; Ferretti et al., 2022; Lee et al., 2024; Sene‐Fiorese et al., 2008; Yu et al., 2023).

Some studies demonstrating significant effects of HIIT on myocardial contractility in different conditions observed that HIIT had a beneficial and superior effect on percentage shortening and contraction and relaxation rates of cardiomyocytes in Wistar rats compared to animals performing continuous exercise of moderate intensity (Kemi et al., 2002, 2007; Wisløff et al., 2002, 2001). Kemi and collaborators (2002). Furthermore, Wisloff and collaborators (2002) demonstrated that HIIT was able to attenuate the reduction in myocardial contractility and significantly improve the sensitivity of myofilaments to Ca^2+^ in infarcted rats.

Regarding intracellular Ca^2+^ handling, few studies have observed the influence of HIIT on cardiac intracellular dynamics (Kemi, 2023). However, the effects of HIIT on myocardial contractility and intracellular Ca^2+^ handling in obesity have not yet been determined. Therefore, the purpose of the current study was to investigate the effects of HIIT on myocardial contractility and intracellular Ca^2+^ handling in obese rats induced by a saturated HFD. The hypothesis is that HIIT enhances myocardial contractility in models of obesity induced by a saturated HFD, as well as promoting an improvement in intracellular Ca^2+^ handling (Ca^2+^ influx and reuptake).

## 2 METHODS

### 2.1 Animal care

Male Wistar rats (*Rattus norvegicus*, *n* = 71), aged 30 days (∼150 g), were procured from the Central Animal Facility of the Federal University of Espírito Santo (Brazil). The animals were individually housed in cages under controlled environmental conditions, including a 12‐h light/dark cycle starting at 06.00 h, ambient temperature (24 ± 2°C), and relative humidity (55 ± 5%). The experimental protocol adhered to the *Brazilian Guideline for the Care and Use of Animals in Teaching or Scientific Research Activities*, published by the National Council for Animal Control and Experimentation (CONCEA – MCT, 2016). Approval for the study was obtained from the Committee of Ethics in the Use of Animals at the Federal University of Espírito Santo (CEUA‐UFES) under protocol 08/2021.

### 2.2 Experimental protocol

The animals underwent a 7‐day acclimatization period and were then randomly assigned to two groups: (a) SD, fed a standard diet, and (b) HFD, fed a high‐fat diet. The SD consisted of 9.47% of calories from fat, 75.81% from carbohydrates and 14.73% from proteins (Nuvilab CR1‐Nuvital, Colombo, Paraná, Brazil). The HFD included a substantial amount of lard, with 45.46% of calories from fat, 40.41% from carbohydrates and 14.13% from protein. All animals had free access to water and chow (40 g/day). To analyze whether dietary‐induced obesity was associated with alterations in nutritional behavior, food consumption (FC) and calorie intake (CI) were measured daily.

Starting from the commencement of the experimental protocol, body mass was recorded weekly, and the nutritional profile was determined as described previously Coelho et al. 2023. The experimental protocol spanned 23 weeks, comprising two experiments: Experiment 1 focused on the induction and maintenance of obesity (week 0–15), while Experiment 2 commenced after the 15^th^ week. In Experiment 2, groups were redistributed, and the HIIT protocol was implemented for 8 weeks.

#### 2.2.1 Experiment 1

The induction of obesity spanned 3 weeks and was marked by an increase in body mass in the HFD group compared to the SD group, signifying the initial phase of obesity. Following this period, rats were sustained in a phase of obesity maintenance for 12 weeks (from week 4 to week 15). At the end of the 15^th^ week, a 95% confidence interval was constructed based on the mean body weight of SD and HFD rats, serving as a separation point (SP) to categorize the sedentary control (C) and sedentary obese (Ob) groups, as demonstrated previously (Coelho et al., 2023).

Subsequently, animals with a body weight above the SP were excluded from the SD group, while animals with a body weight below the SP were excluded from the HFD group. These animals were reallocated in other studies from our laboratory. Thus, two homogeneous groups, SD (*n* = 25) and HFD (*n* = 23), were formed.

#### 2.2.2 Experiment 2

Following the establishment of the SP, the SD and HFD groups were systematically randomized and redistributed based on the absence and/or presence of HIIT into four groups: (1) sedentary control (C, *n* = 12), (2) control subjected to HIIT (CHIIT, *n* = 12), (3) sedentary obese (Ob, *n* = 13), and (4) obese subjected to HIIT (ObHIIT, *n* = 10).

### 2.3 FC, caloric intake and feed efficiency

Feed efficiency (FE; %) was calculated by dividing the total weight gain of the animals (g) by the total caloric intake (kcal) multiplied per 100 (Coelho et al., 2023). Calorie intake was calculated by weekly FC multiplied by the caloric value of each diet (g kcal).

### 2.4 HIIT protocol

#### 2.4.1 Familiarization

At week 15, all groups were familiarized with an adaptation period on the treadmill at low intensity at speed of 3 m/min for 10 min per day, five consecutive days. All animals performed the ${\dot{V}}_{O_{2}\max}$ and progressive physical performance tests at least 48 h after the last familiarization session with the treadmill. Subsequent to the ${\dot{V}}_{O_{2}\max}$ test, animals in the CHIIT and ObHIIT groups were then subjected to HIIT for 8 weeks.

#### 2.4.2 HIIT protocol

The HIIT protocol employed an adapted high‐intensity interval running regimen (Mendes et al., 2022), conducted on a specialized treadmill designed for rats (Bonther, Ribeirão Preto, SP, Brazil), 5 days a week. The intensity ranged from 85% to 100% of ${\dot{V}}_{O_{2}\max}$, with a duration progression from 15 to 20 min, implemented over an 8‐week period. The initiation of HIIT occurred in week 16 of the experimental protocol, with a division into two protocols incorporating a progression in training duration. HIIT sessions were conducted between 11.00 h and 13.00 h, with training intensity determined by a ${\dot{V}}_{O_{2}\max}$ test.

**FIGURE 1:**
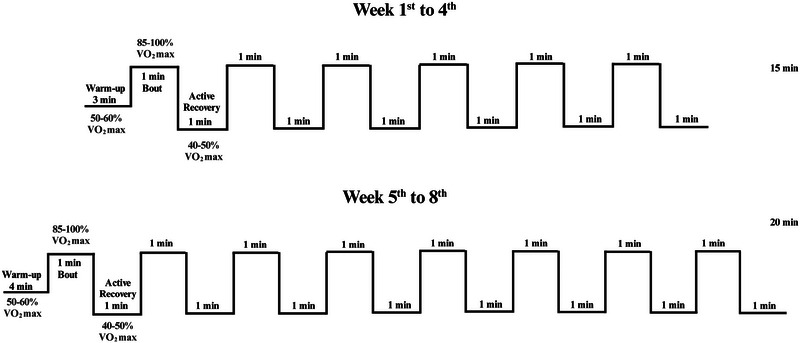
High‐intensity interval training protocol. The control group subjected to high‐intensity interval training (CHIIT) and obese group submitted to high‐intensity interval training (ObHIIT) engaged in daily sessions, five times a week, lasting 15 min each session. The protocol included a 3‐min warm‐up at 50–60% of ${\dot{V}}_{O_{2}\max}$, followed by six 1‐min bouts starting from the 1st week at 85% of ${\dot{V}}_{O_{2}\max}$, progressing up to 100% of ${\dot{V}}_{O_{2}\max}$ by the 4th week. Active recovery was implemented with six 1‐min sessions at 40–50% of ${\dot{V}}_{O_{2}\max}$. In the 5^th^ week, the CHIIT and ObHIIT groups continued daily sessions, five times per week, extended to 20 min each. The extended protocol involved a 4‐min warm‐up at 50–60% of ${\dot{V}}_{O_{2}\max}$, followed by eight 1‐min bouts starting from the 5^th^ week at 85% of ${\dot{V}}_{O_{2}\max}$, progressing to 100% of ${\dot{V}}_{O_{2}\max}$ by the 8th week. Active recovery consisted of eight 1‐min sessions at 40–50% of ${\dot{V}}_{O_{2}\max}$.

During the initial 4 weeks of the HIIT protocol (from the 1st to the 4th week), daily sessions (5 days/week) consisted of a 3‐min warm‐up at 50–60% of ${\dot{V}}_{O_{2}\max}$, followed by six bouts: 1‐min sessions at 85−100% of ${\dot{V}}_{O_{2}\max}$, interspersed with 1 min of active recovery (at 40–50% of ${\dot{V}}_{O_{2}\max}$), resulting in sessions lasting a total of 15 min (Figure 1). In the subsequent 5th to 8th weeks of the HIIT protocol, sessions (5 days/week) were extended to 20 min. These sessions comprised a 4‐min warm‐up at 50–60% of ${\dot{V}}_{O_{2}\max}$, followed by eight bouts: 1‐min sessions at 85−100% of ${\dot{V}}_{O_{2}\max}$, interspersed with 1 min of active recovery (at 40–50% of ${\dot{V}}_{O_{2}\max}$), as illustrated in Figure 1.

### 2.5 Assessment of ${\dot{V}}_{O_{2}\max}$ and progressive physical performance test

The assessment of ${\dot{V}}_{O_{2}\max}$ in animals was conducted using an adapted progressive treadmill protocol (Mendes et al., 2022; Wisløff et al., 2002). Throughout the HIIT protocol, the animals performed three ${\dot{V}}_{O_{2}\max}$ tests. The first test occurred 48 h before the initiation of the HIIT protocol, the second after 4 weeks to fine‐tune the intensity of the HIIT protocol and establish a new maximum running intensity, and the third test at the conclusion of the experimental protocol. The ${\dot{V}}_{O_{2}\max}$ analysis protocol comprised three stages: first stage: 10‐min period with the animal standing in the metabolic chamber for baseline ${\dot{V}}_{O_{2}}$ analysis; second stage: 5‐min warm‐up with an intensity of 3 m/min and; third stage: after 5 min of warm‐up, the treadmill running velocity was increased every 3 min by 3 m/min until the definition of ${\dot{V}}_{O_{2}\max}$. The criteria for interrupting the test were: (1) when there was an increase in treadmill speed, but ${\dot{V}}_{O_{2}}$ reached a plateau with no increase greater than 5%; (2) the ${\dot{V}}_{CO_{2}}$ and ${\dot{V}}_{O_{2}}$ (RER) ratio exceeded 1.05; and (3) the animal stopped running and remained in the shock bay for 15 s.

${\dot{V}}_{O_{2}\max}$, expired carbon dioxide (${\dot{V}}_{CO_{2}}$), and respiratory exchange ratio (RER) were continuously monitored during testing using an O_2_ and CO_2_ analyser. The gas monitoring system and treadmill were connected to a computer and evaluated by software (Panlab—Harvard Apparatus, Holliston, MA, USA). This software allowed the collection of various variables: distance covered (m), maximum speed (m/min), time (min), baseline ${\dot{V}}_{O_{2}}$, ${\dot{V}}_{O_{2}\max}$, and the delta (Δ) of ${\dot{V}}_{O_{2}}$. The delta (Δ) of ${\dot{V}}_{O_{2}}$ was calculated using the formula: (${\dot{V}}_{O_{2}\max}$ – ${\dot{V}}_{O_{2}}$ baseline)/${\dot{V}}_{O_{2}}$ baseline multiplied by 100 and expressed as a percentage. To obtain max ${\dot{V}}_{O_{2}}$, the values of the last stage reached by the animal were used, and ${\dot{V}}_{O_{2}}$ baseline was extracted in the first 10 min before starting the treadmill movement. The distance covered, time, and maximum running speed achieved were determined as the maximum values reached at the end of the test for each variable.

### 2.6 Glucose tolerance and insulin tolerance tests

Blood collections from the caudal artery were performed following an 8‐h fasting period and subsequent intraperitoneal administration of 50% glucose (Sigma‐Aldrich, St Louis, MO, USA), equivalent to 2 g/kg for the tests. For the glucose tolerance test (GTT), blood samples were collected at time points (considered baseline) 30, 60, 90 and 120 min after glucose infusion.

Glucose levels were measured using a portable glucometer from the Accu‐Chek Go Kit brand (Roche Diagnostics Brazil Ltda, São Paulo, Brazil). Glucose intolerance in these animals was assessed by curve profile and glucose area (area under the curve, AUC) (Cassidy et al., 2017; Matthews et al., 1985).

To evaluate insulin sensitivity in vivo, the insulin tolerance test (ITT) was performed. The first collection was carried out before insulin administration (basal) following an 8‐h fasting period. Subsequently, 0.5 IU/kg BW of regular insulin (Novolin 100U/mL, Novo Nordisk, Bagsvaerd, Denmark) was administered. Blood glucose levels were measured at times 0 (baseline), 30, 60 and 90 min after insulin administration. The decay constant (kITT, %/min.) was then calculated from the linear regression of the blood glucose concentrations obtained during the test (Matthews et al., 1985; Mendes et al., 2022).

### 2.7 Systemic insulin resistance

The assessment of resistance to insulin action was analysed using the Homeostatic Model Assessment of Insulin Resistance (HOMA‐IR) and the Homeostatic Model Assessment of the Insulin Secretion Capacity of Pancreatic Beta Cells (HOMA‐β) indexes, based on fasting serum glucose and insulin concentrations. HOMA‐IR was determined by the following formula: insulin concentration (μU/mL) multiplied by glucose levels (mM/L) divided by 22.5 (Cassidy et al., 2017); HOMA‐β was determined by the following formula: insulin concentration (μU/mL) multiplied by 20, divided by glucose levels (mM/L), and then 3.5 subtracted (Cassidy et al., 2017).

### 2.8 Euthanasia

At the conclusion of the experimental protocol (23 weeks), following an 8‐h fasting period, the animals were injected with sodium heparin (1000 U/kg; i.p.). Subsequently, they were anesthetized and sedated with ketamine (70 mg/kg/i.p.) and xylazine (10 mg/kg/i.p.). In cases where animals still exhibited signs of nociceptive reflex after anaesthetic induction, an anaesthetic overdose (lethal dose) was administered, consisting of three times the doses of ketamine hydrochloride and xylazine hydrochloride used during the animal's anaesthetic induction (Favoretto et al., 2019). Following euthanasia, the animals were submitted to a median thoracotomy to collect blood and tissue samples.

### 2.9 Lipid and hormonal profiles

To analyse the lipid profile and hormonal levels, blood samples were collected in Falcon tubes, centrifuged at 3000 × *g* and 4000 rpm for 10 min (Eppendorf Centrifuge 5804‐R, Hamburg, Germany), and stored in a freezer at −80°C. Plasma concentrations of total cholesterol and high‐density lipoprotein (HDL) were determined using specific kits (Bioclin, Belo Horizonte, Brazil and Synermed do Brasil Ltda, São Paulo, Brazil) and analysed by the automated biochemical analyser BS‐200 (Mindray, Mahwah, NJ, USA).

Hormonal concentrations of glucagon and insulin were determined by the enzyme‐linked immunosorbent assay using specific kits (Linco Research Inc., St Louis, MO, USA) following the manufacturer's instructions. Reading was performed using a microplate reader (Spectra MAX 190, Molecular Devices, San Jose, CA, USA).

### 2.10 Cardiac remodelling

The process of cardiac remodelling was analysed through structural, histological and functional studies, as described below. The cardiac injury marker was assessed by measuring the creatine kinase myocardial band (CK‐MB) using a specific kit (Bioclin, Belo Horizonte, Brazil) and measured with an automated biochemical analyser BS‐200 (Mindray).

Additionally, a cross‐sectional area (CSA) analysis was performed using samples from the left ventricle (LV). After a median thoracotomy, tissue fragments were placed in a paraformaldehyde solution (4%, pH 7.4). Subsequently, the tissues were transferred to an ethanol solution (70%, 80%, 90%, and 100%) and included in the paraffin block. The 5 μm thick histological sections were stained on a slide with haematoxylin–eosin (H&E) solution and observed at ×40 magnification with a microscope (AX70, Olympus Optical Co., Hamburg, Germany), coupled to a camera that digitizes the images. These images were then analysed by Image Pro‐plus software (Media Cybernetics, Silver Spring, MD, USA).

To calculate myocyte CSA, 50–70 cells were measured per tissue. The myocytes analysed were located in the subendocardial layer of the muscular wall. The CSA of the samples (μm^2^) was used as indicators of cell size, characterizing the presence or absence of left ventricular hypertrophy (Wisløff et al., 2002, 2001).

### 2.11 Myocardial interstitial collagen

The determination of the interstitial collagen fraction (%) was carried out using the Picrosirius Red technique, with perivascular collagen being excluded. The LV fragments were transferred to 70% ethanol diluted in water, included in paraffin blocks and stained with Picrosirius Red. Quantification of interstitial collagen was performed using 15 fields per fragment.

The tissue components were identified according to the level of staining: red, collagen fibres; yellow, myocytes; and white, interstitial space. The histological sections were magnified ×40 using a microscope (Olympus AX70) coupled to a camera that digitized the images. Analyses were performed using Image Pro‐plus software.

### 2.12 Analysis of muscle fibre diameter and adipocyte area

The gastrocnemius and visceral fat pad tissue fragments were fixed in 4% paraformaldehyde for 48 h. Subsequently, they underwent dehydration using an increasing alcohol gradient (70%, 80%, 90% and 100%) and were then embedded in paraffin.

The samples were cut using a microtome with 5 μm thickness. Slides were stained with H&E and observed at ×40 magnification using a microscope (Olympus AX70), coupled to a camera that digitized the images. The digitized images were then analysed using Image Pro‐plus software.

Transverse sections (∼15 fields per animal) were employed for analysing muscle fibre diameter (Mendes et al., 2022). The cell area of adipocytes was measured, and the calculation was based on the average value of the area across all measured fields (Melo et al., 2019) for each group. The number of adipocytes was determined following the method of Gibert‐Ramos et al. (2020).

### 2.13 Isolation of cardiac myocytes

At the end of the experimental period (23 weeks), the animals were euthanized and subjected to median thoracotomy. The hearts were quickly removed and transferred to a Petri dish containing digestion solution (Digestion Buffer; DB), with a modified isolation digestion buffer solution (DB), a calcium‐free solution containing 0.1 mM ethylene glycol‐bis (ß‐aminoethyl ether)‐N, N, N′, N′‐tetraacetic acid (EGTA) and N‐[2‐hydro‐ethyl]‐piperazine‐N′‐[2‐ethanesulfonic acid] (HEPES). The DB solution consists of a basic solution with ultrapure water (milli‐Q), with the following composition (mM): NaCl (130); MgCl_2_ (1.0); KCl (5.4); HEPES (25); glucose (22); NaH_2_PO_4_ (0.33); pH 7.39.

After cleaning the heart, the tissue was weighed in a beaker containing 20 mL of the DB + EGTA + HEPES solution. The aorta artery was cannulated using the Langendorff technique and the heart was perfused with a DB + EGTA + HEPES solution in constant flow to clean the coronary vessels for a period of 2–3 min.

After carrying out this procedure, the heart was perfused with an enzyme solution containing DB solution, collagenase (25 mg) and Ca^2+^ (1 mM) for a period of 10–15 min. The solutions used in this process were continuously aerated with 95% O_2_ and 5% CO_2_ and kept in a water bath at 37.5°C.

In the perfusion period, the atria were removed, and the heart was cut in a Petri dish containing DB solution + albumin + collagenase + Ca^2+^ (1 mM). The heart fragments (pellet) were lightly resuspended in a beaker with a Pasteur pipette for 2 min in a water bath at 37.5°C. Then, the cells were dissociated, resuspended and filtered. After 10 min, the supernatant was removed, keeping the pellet, and then solutions of DB + albumin + 1 mM Ca^2+^ were added. The step described above was repeated twice, but with increments in the volume of Ca^2+^ (1.6 and 3.12 μL).

At each step, the tube containing the cells and solutions was kept at rest for approximately 10 min and the supernatant was discarded. After completing these steps (two changes of solution) and removing the supernatant, the stock solution (Tyrode) with the following composition (mM) was added to the pellet: NaCl (140); HEPES (10); NaH_2_PO_4_ (0.33); MgCl_2_ (1); KCl (5); CaCl_2_ (1.8); and glucose (10). The solutions used in this process were previously heated to 37.5°C.

#### 2.13.1 Cardiomyocyte contraction

Cellular contraction was measured using the cardiomyocyte length change technique, using an edge detection system coupled to an inverted microscope (IonOptix, Westwood, MA, USA) with a ×40 objective lens (Nikon Eclipse – TS100, Melville, NY, USA). The cardiomyocytes were placed in an experimental camera with a glass base, bathed in Tyrode solution and viewed on a monitor using a camera (Myocam, IonOptix, 240 Hz) coupled to the microscope with an image detection program (Ionwizard, IonOptix).

Cardiomyocytes were stimulated at a frequency of 1 Hz for 5 ms and a voltage of 15 V, using a pair of steel electrodes and an electrical field stimulator (Myopacer, IonOptix). Contractile measurements were performed on isolated cardiomyocytes that presented the following conditions: well‐defined sarcomere borders and striations, relaxed at rest and without presenting voluntary contractions. The parameters of percentage shortening, maximum shortening and relaxation rates (μm/s), times to 50% of PS and relaxation (ms) were analysed.

#### 2.13.2 Analysis of intracellular Ca^2+^ handling

Cardiomyocytes were incubated with the fluorescent Ca^2+^ indicator Fura‐2 AM (Thermo Fisher Scientific, Waltham, MA, USA). To evaluate Ca^2+^ handling, cells were loaded with 1 μM Fura‐2 AM for 20 min at room temperature.

Before image acquisition, myocytes were electrically stimulated at 1 Hz (Myopacer 100, IonOptix) with the following settings: duration: 2 ms; continuous biphasic pulse stimulation; voltage: adjusted to 120% of the threshold voltage that induces Ca^2+^ handling. Fluorescence images were obtained using 340 and 380 nm excitation, and fluorescence intensity emission detected at ∼510 nm. The parameters of Ca^2+^ handling amplitude (*F*/*F*_0_), Ca^2+^ systolic (nM), Ca^2+^ diastolic (nM), Ca^2+^ release rate (μm/s), and Ca^2+^ reuptake rate (μm/s) were analysed. In addition, the ratios of systolic Ca^2+^ to percentage shortening and systolic Ca^2+^ to maximum shortening rate were obtained to evaluate the responsiveness of myofilaments to Ca^2+^.

### 2.14 Statistical analysis

The general characteristics, comorbidities, cardiorespiratory fitness tests, macro and functional analysis of the experimental groups were expressed as means ± standard deviation (SD). All datasets were evaluated for distribution using the D'Agostino and Pearson test. Comparisons among the four groups were assessed using two‐way analysis of variance (ANOVA) for independent samples, followed by Tukey's *post‐hoc* test. The significance level considered for all variables was 5%. The statistical analyses and graphics were conducted using GraphPad Prism 9.0 software (GraphPad, Boston, MA, USA).

## 3 RESULTS

**FIGURE 2:**
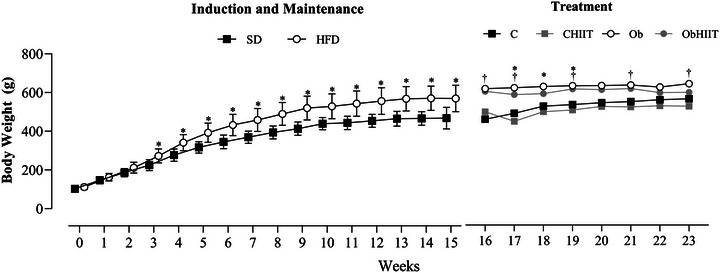
Effect of HIIT on the evolution of body weight (g) in the different groups assessed during the 8‐week treatment period. Values are expressed as means ± standard deviation. *P* < 0.05: *SD versus HFD (induction and maintenance to obesity); and HIIT treatment, *P* < 0.05: *C versus Ob; ^†^CHIIT versus ObHIIT. The analysis utilized two‐way ANOVA for repeated measures, complemented with Tukey's *post‐hoc* test. C, sedentary control (*n* = 12); CHIIT, control submitted to high‐intensity interval training (*n* = 12); Ob, sedentary obese (*n* = 13); ObHIIT, obese submitted to high‐intensity interval training (*n* = 10).

Figure 2 presents body weight data during the 23‐week experimental protocol. At the start of the protocol, the SD and HFD groups exhibited similar body weight. However, during the obesity induction period (week 3), the HFD group showed a statistically significant increase in body weight compared to the SD group (*P* < 0.0001), marking the initial stage of obesity. Subsequently, HFD animals maintained a significantly higher body weight than the SD group during the obesity maintenance period (weeks 4–15) (Figure 2).

Following the induction and maintenance of obesity, animals were redistributed based on the presence or absence of HIIT. Throughout the HIIT protocol (weeks 16–23), it was observed that from the 16^th^ week onwards, HIIT did not lead to a reduction in body weight in the groups undergoing the physical training protocol (CHIIT and ObHIIT) compared to the sedentary groups (Figure 2). When comparing the trained animals, it was noted that in some weeks of treatment, HIIT resulted in a decrease in body weight, as the CHIIT group exhibited a lower body mass than the ObHIIT group (weeks 16, 17, 19, 21 and 23).

Table 1 depicts the nutritional profile and body composition data. HIIT did not induce significant changes in adiposity within the experimental groups. However, concerning nutritional parameters, the Ob group exhibited lower FC compared to group C. Similar results were observed in the trained groups (CHIIT > ObHIIT). Concerning caloric intake, group C demonstrated statistically higher values than group Ob. No significant statistical differences were observed among groups for feed efficiency. Additionally, there were no significant differences in nutritional parameters between the Ob and ObHIIT groups.

**TABLE 1:** Nutritional profile and body composition of the experimental groups after HIIT protocol.

Variable	Group			
	C	CHIIT	Ob	ObHIIT
Food consumption (g/day)	25.3 ± 1.59	23.7 ± 1.91	17.8 ± 1.45*	18.7 ± 2.38^∑^
Caloric intake (kcal/day)	96.4 ± 6.06	93.4 ± 7.48	86.2 ± 7.00*	90.4 ± 11.5
Feed efficiency (%)	1.05 ± 0.39	0.49 ± 0.49	0.58 ± 0.51	0.79 ± 0.96
IBW (g)	466 ± 65.2	470 ± 46.4	562 ± 72*	560 ± 28 ^∑^
FBW (g)	531 ± 54	500 ± 38.8	595 ± 76.3	606 ± 53.2 ^∑^
Body weight gain (g)	65.4 ± 25.6	30.4 ± 29.5	32.2 ± 28.2	46.7 ± 64.8
Epididymal fat pad (g)	10.0 ± 3.3	7.05 ± 3.0	11.4 ± 3.8	11.1 ± 2.3^∑^
Visceral fat pad (g)	8.03 ± 3.8	5.69 ± 1.4	8.03 ± 3.5	9.7 ± 3.8^∑^
Retroperitoneal fat pad (g)	13.2 ± 4.6	10.4 ± 2.5	16.5 ± 9.0	19.0 ± 8.1^∑^
Total body fat (g)	31.3 ± 11.1	23.1 ± 6.2	36.0 ± 15.5	39.7 ± 13.3^∑^
Adiposity index (%)	5.78 ± 1.6	4.58 ± 1.2	6.00 ± 1.6	7.04 ± 2.3^∑^

IBW, initial body weight; FBW, final body weight; C, sedentary control; CHIIT, control submitted to high‐intensity interval training; Ob, sedentary obese; ObHIIT, obese submitted to high‐intensity interval training. *p < 0.05 C *versus* Ob; ^∑^ CHIIT *versus* ObHIIT. Two‐way ANOVA for independent samples, complemented with Tukey's *post‐hoc* test.

Regarding initial body weight, group C exhibited significantly lower body weight compared to group Ob (*P* = 0.0006). Concerning the trained groups, the results indicate that the ObHIIT group had statistically greater body weight compared to the CHIIT group (*P* = 0.003). Additionally, the ObHIIT group had a greater final body weight compared to the CHIIT group (*P* = 0.001). However, the CHIIT group had lower body fat, adiposity index, and epididymal, visceral and retroperitoneal fat pads when compared to the ObHIIT group. Comparing CHIIT versus C, the adiposity parameters were numerically reduced in CHIIT, but the reduction was not significant. Nevertheless, looking at the data of HIIT in obesity, the results showed that HIIT did not reduce final body weight or adiposity parameters in the obese group, suggesting that this exercise could be effective in control conditions but not in obesity.

Table 2 displays the assessments of maximum oxygen consumption and progressive physical performance tests conducted during the treatment period of the experimental protocol. No differences were found in ${\dot{V}}_{O_{2}}$ baseline and ${\dot{V}}_{O_{2}\max}$ among the groups in the first and second tests. However, these parameters were higher in the trained groups (CHIIT and ObHIIT) after the third test when compared to their respective sedentary groups (${\dot{V}}_{O_{2}\max}$: ObHIIT vs. Ob, Δ 60%) and (${\dot{V}}_{O_{2}\max}$: CHIIT vs. C, Δ 43.5%). Regarding performance parameters, the ObHIIT group exhibited significantly higher speed values than Ob (*P* < 0.05) in the second and third tests. In relation to CHIIT, statistically higher speed values were observed in the second and third tests when compared to C (*P* < 0.05). Concerning distance, the results showed that the trained groups (CHIIT vs. C, 55.3% and ObHIIT vs. Ob, 49.4%) presented significantly higher values compared to their respective sedentary groups (*P* < 0.05) in the third test. The other parameters showed no statistical difference.

**TABLE 2:** Effect of HIIT on the parameters of ${\dot{V}}_{O_{2}}$ during the tests performed.

Variable	Test 1				Test 2				Test 3			
	C	CHIIT	Ob	ObHIIT	C	CHIIT	Ob	ObHIIT	C	CHIIT	Ob	ObHIIT
${\dot{V}}_{O_{2}}$ baseline (mL.kg‐0.75.min^−1^)	25 ± 4	25 ± 3	24 ± 4	26 ± 3	25 ± 4	28 ± 4	26 ± 4	28 ± 5	27 ± 4	34 ± 4^§^	25 ± 5	31 ± 6^#^
${\dot{V}}_{O_{2}\max}$ (mL.kg‐0.75.min^−1^)	38 ± 6	38 ± 6	34 ± 8	39 ± 8	43 ± 15	53 ± 7	45 ± 9	50 ± 9	46 ± 10	66 ± 10^§^	40 ± 9	64 ± 10^#^
Δ ${\dot{V}}_{O_{2}}$ (%)	56 ± 29	54 ± 19	42 ± 40	46 ± 25	70 ± 50	90 ± 23	72 ± 26	79 ± 33	76 ± 51	98 ± 32	61 ± 46	111 ± 66
RER (${\dot{V}}_{CO_{2}}$/${\dot{V}}_{O_{2}}$)	1.02 ± 0.20	1.04 ± 0.21	1.02 ± 0.36	1.03 ± 0.26	0.97 ± 0.25	1.07 ± 0.41	0.86 ± 0.06	0.96 ± 0.19	1.00 ± 0.38	1.02 ± 0.19	0.95 ± 0.20	0.95 ± 0.18
Speed (m/min)	28 ± 7	29 ± 3	27 ± 5	29.5 ± 48	28 ± 6	33 ± 6	27 ± 8	34 ± 7^#^	31 ± 5	41 ± 7^§^	28 ± 6	36 ± 3^#^
Distance (m)	275 ± 112	277 ± 63	260 ± 84	287 ± 73	274 ± 113	353 ± 134	256 ± 132	355 ± 140	309 ± 104	480 ± 150^§^	259 ± 119	386 ± 47^#^
Time (min)	26.3 ± 3.5	27.3 ± 3.7	26.9 ± 3.4	27.4 ± 3.8	24.8 ± 4.7	25.9 ± 3.1	25.0 ± 6.3	26.8 ± 5.3	26.7 ± 5.7	30.7 ± 5.6	23.0 ± 6.7	27.5 ± 3.5

*Note*: Effect of HIIT on the parameters of baseline and maximal oxygen consumption (${\dot{V}}_{O_{2}}$). Speed, distance, and time during the tests were performed. Values expressed as means ± standard deviation. *P* < 0.05: ^§^C versus CHIIT; ^#^Ob versus ObHIIT. Two‐way ANOVA for repeated measures complemented with Tukey's *post‐hoc* test. Groups: C, sedentary control (*n* = 12); CHIIT, control submitted to high‐intensity interval training (*n* = 12); Ob, sedentary obese (*n* = 13); ObHIIT, obese submitted to high‐intensity interval training (*n* = 10). Δ ${\dot{V}}_{O_{2}}$, ${\dot{V}}_{O_{2}\max}$ – ${\dot{V}}_{O_{2}}$ baseline)/${\dot{V}}_{O_{2}}$ baseline multiplied by 100; RER, respiratory exchange ratio.

**FIGURE 3:**
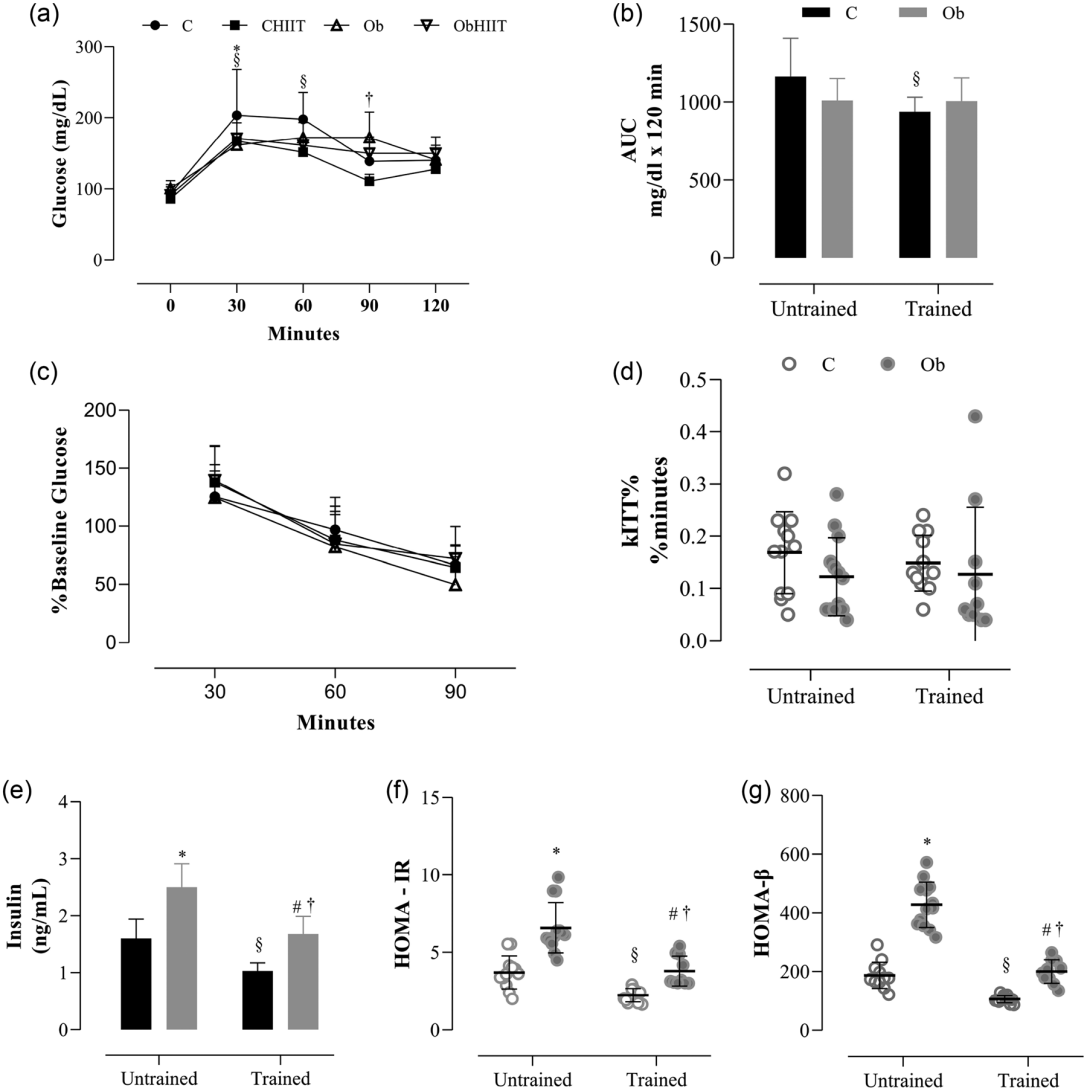
Effects of HIIT on the glycaemic, hormonal and metabolic profile in the different groups assessed during the 8‐week treatment period. (a, b) Plasma glucose levels during oral glucose tolerance test; AUC, area under the curve for glucose. (c, d) Plasma glucose levels during insulin tolerance test; kITT, decay constant. (e) Plasma insulin levels. (f, g) HOMA‐IR (homeostatic model assesses insulin resistance) and HOMA‐β (homeostatic model assesses the insulin secretion capacity of pancreatic β‐cells). Values are expressed as means ± standard deviation. *P* < 0.05: *C versus Ob; ^§^C versus CHIIT; ^#^Ob versus ObHIIT; ^†^CHIIT versus ObHIIT. The analysis utilized two‐way ANOVA for independent samples, complemented with Tukey's *post‐hoc* test. C, sedentary control (*n* = 12); CHIIT, control submitted to high‐intensity interval training (*n* = 12); Ob, sedentary obese (*n* = 13); ObHIIT, obese submitted to high‐intensity interval training (*n* = 10).

Figure 3 shows the effects of HIIT on glucose, hormonal and metabolic profile parameters. Regarding the GTT (Figure 3a), the results show that there was no statistical difference at the baseline moment. However, a statistical difference was observed with lower glucose levels at moment 30 between the CHIIT and Ob groups compared to C. After 60 min, C rats had statistically higher glucose compared to the CHIIT group. Additionally, the CHIIT group promoted a reduction in glucose levels after 90 min, compared to the ObHIIT group. At 120 min, there was no statistical difference between the groups.

Regarding AUC, HIIT was effective in reducing the AUC in C, as the CHIIT presented lower glucose values compared to C (Figure 3b). However, no statistical differences were observed for AUC in relation to the other groups. Considering percentage baseline glucose (Figure 3c), which shows the reduction in blood glucose after insulin administration, no statistical difference was observed among groups, or for kITT% (Figure 3d). Additionally, the Ob group presented statistically higher insulin levels compared to the C group. Comparing the trained groups with their respective sedentary groups, there was a reduction in insulin levels in CHIIT and ObHIIT compared to C and Ob rats. Regarding only the trained groups, the results show that the ObHIIT group presented statistically higher levels compared to the CHIIT group (*P* = 0.0002) (Figure 3e). There was a statistical difference for glucagon (C, 6.07 ± 1.46 ng/dL; CHIIT, 6.63 ± 1.41 ng/dL; Ob, 4.90 ± 1.35 ng/dL; ObHIIT, 5.56 ± 1.01 ng/dL; data not shown).

Considering the HOMA‐IR (Figure 3f) and HOMA‐β (Figure 3g) analysis, the results showed that the Ob group presented statistically higher values compared to the C group. Comparing the trained groups with their respective control groups, we observed that CHIIT and ObHIIT presented a reduction in HOMA‐IR and HOMA‐β compared to C and Ob, respectively. In relation to the trained groups, the results show that the ObHIIT group presented higher values compared to the CHIIT group. For the lipid profile, the results showed that the CHIIT group had statistically higher serum levels of HDL‐c compared to the ObHIIT group (*P* = 0.022). Regarding the parameters of total cholesterol (C, 60.5 ± 10.6 mg/dL; CHIIT, 55.3 ± 17.7 mg/dL; Ob, 62.3 ± 16.3 mg/dL; ObHIIT, 60.0 ± 11.2 mg/dL; *P* > 0.05) and CK‐MB (C, 309 ± 119 U/L; CHIIT, 182 ± 51 U/L; Ob, 419 ± 230 U/L; ObHIIT, 236 ± 145 U/L; data not shown), no statistical differences were found.

**FIGURE 4:**
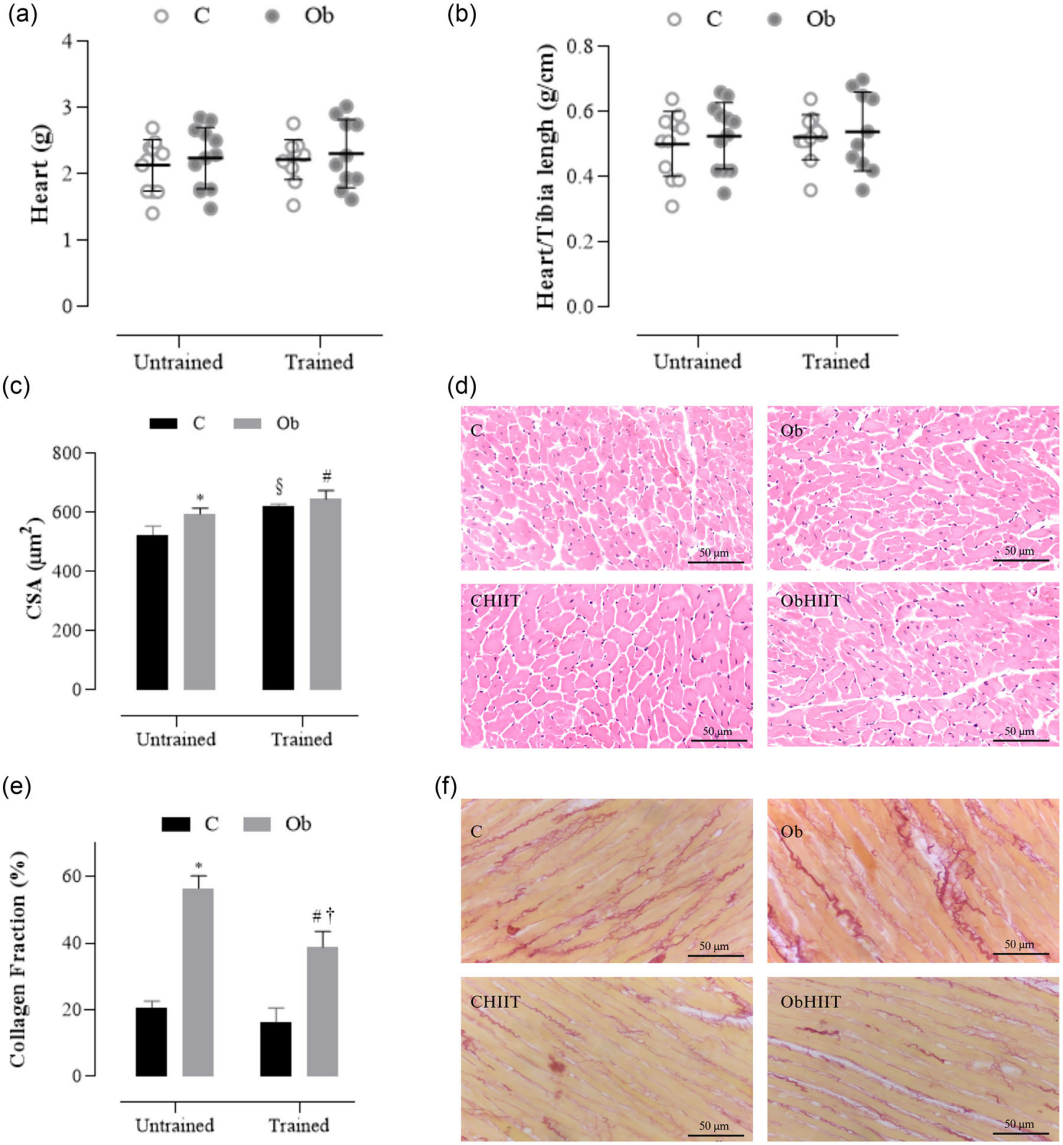
Effects of HIT on macroscopy and microscopy of the heart. (a, b) Heart mass (g) and heart/tibia length ratio (g/cm). (c, d) Left ventricle (LV) cross sectional area (CSA, μm^2^) and representative H&E slides 50 μm. (e, f) LV collagen fraction (%) and representative Picrosirius Red slides 50 μm. Groups were evaluated after the end of experimental protocol (23 weeks). Values are expressed as means ± standard deviation. *P* < 0.05: *C versus Ob; ^§^C versus CHIIT; ^#^Ob versus ObHIIT; ^†^CHIIT versus ObHIIT. The analysis utilized two‐way ANOVA for independent samples, complemented with Tukey's *post‐hoc* test. C, sedentary control (*n* = 4); CHIIT, control subjected to high‐intensity interval training (*n* = 5); Ob, sedentary obese (*n* = 5); ObHIIT, obese subject to high‐intensity interval training (*n* = 4).

Figure 4 depicts the impact of HIIT on morphological parameters and collagen deposition. No statistical difference was observed in the parameters of heart mass and heart/tibia length ratio among the groups (Figure 4a, b). Regarding the cardiomyocyte CSA, the results indicate that the Ob group exhibited an elevation in CSA compared to the C group (an increase of 18.5%), signifying cardiomyocyte hypertrophy (Figure 4c, d). Additionally, the trained groups demonstrated higher CSAs in comparison to their respective sedentary groups (Figure 4c, d). Concerning the collagen fraction (Figure 4e, f), the results reveal that the Ob group exhibited higher collagen deposition than the C group. It is noteworthy that HIIT only ameliorated but did not prevent collagen accumulation in cardiac tissue in the ObHIIT group. Furthermore, the ObHIIT group displayed a higher fraction of interstitial collagen compared to the CHIIT group (*P* < 0.0001), indicating an obesity‐related effect.

**FIGURE 5:**
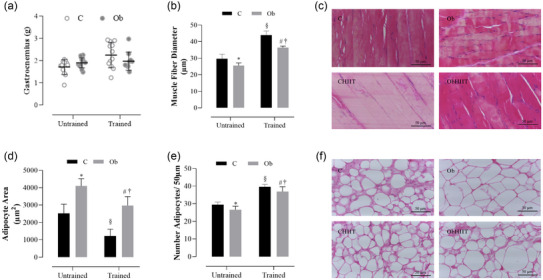
Effects of HIIT on muscle fibre diameter (gastrocnemius muscle) and adipocyte size (visceral adipose tissue). (a) Gastrocnemius mass (g). (b, c) Muscle fibre diameter (gastrocnemius, μm) and representative H&E slides 50 μm. (d) Adipocyte size (μm^2^). (e, f) Number of adipocytes per area and representative H&E slides 50 μm. Values expressed as means ± standard deviation. *P* < 0.05: *C versus Ob; ^§^C versus CHIIT; ^#^Ob versus ObHIIT; ^†^CHIIT versus ObHIIT. Two‐way ANOVA for independent samples, complemented with Tukey's *post‐hoc* test. C, sedentary control (*n* = 4); CHIIT, control submitted to high‐intensity interval training (*n* = 5); Ob, sedentary obese (*n* = 5); ObHIIT, obese submitted to high‐intensity interval training (*n* = 4).

Figure 5 illustrates the effects of HIIT on histological muscular and adiposity parameters. No statistical difference was observed in gastrocnemius muscle mass among the groups (Figure 5a). However, the gastrocnemius fibre diameter was reduced in obesity (Figure 5b). Additionally, statistical differences were found in the gastrocnemius fibre diameter in trained groups when compared to their respective sedentary groups. It was observed that both CHIIT and ObHIIT groups had a larger muscle fibre diameter. When comparing the trained groups, it was noted that the CHIIT group had a larger muscle fibre diameter compared to the ObHIIT group.

In the analysis of the area of adipocytes in visceral adipose tissue, the results indicate that the Ob group presented a higher adipocyte area and a lower number of adipocytes compared to the C group, respectively (Figure 5d, e). Concerning groups C and CHIIT, it was observed that group C had a larger adipocyte area when compared to CHIIT, but the number of adipocytes was reduced. However, HIIT was able to reduce the adipocyte area without diminishing the number of adipocytes in obesity (ObHIIT versus Ob). Regarding the trained groups, CHIIT and ObHIIT, it was observed that the ObHIIT group had a larger adipocyte area and a reduced number of adipocytes when compared to the CHIIT group (Figure 5d, e).

**FIGURE 6:**
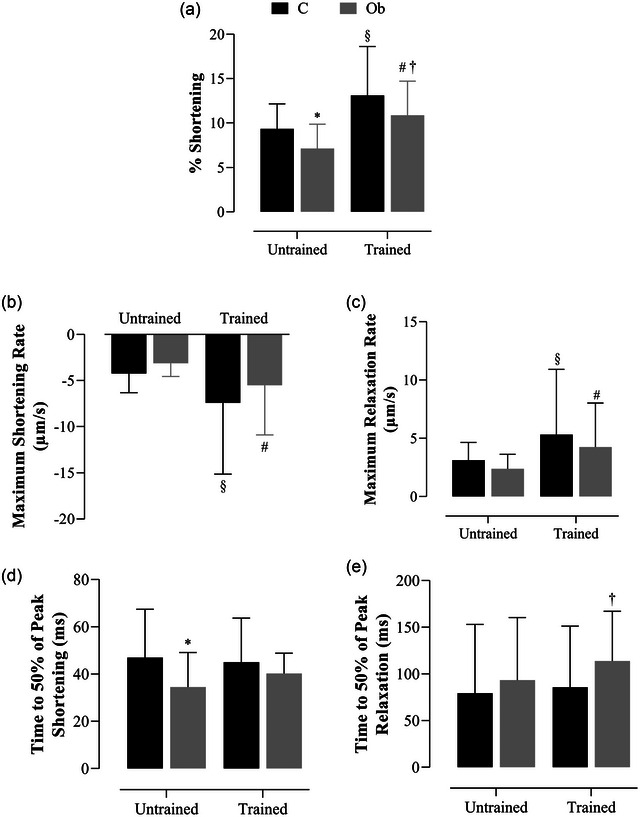
Effects of HIIT on cardiomyocyte contractility parameters, stimulated at 1 Hz. (a) Shortening expressed as percentage of resting sarcomere length. (b, c) Maximum rate of shortening and relaxation (μm/s). (d, e) Times to 50% shortening and relaxation (ms). Values expressed as means ± standard deviation. *P* < 0.05: *C versus Ob; ^§^C versus CHIIT; ^#^Ob versus ObHIIT; ^†^CHIIT versus ObHIIT. Two‐way ANOVA for independent samples, complemented with Tukey's *post‐hoc* test. C, sedentary control (*n* = 8, cells = 78); CHIIT, control submitted to high‐intensity interval training (*n* = 7, cells = 87); Ob, sedentary obese (*n* = 6, cells = 65); ObHIIT, obese submitted to high‐intensity interval training (*n* = 6, cells = 70).

Figure 6 illustrates the effects of HIIT on the contractile parameters of isolated cardiomyocytes. The results showed the Ob rats presented a reduction in percentage shortening when compared with C rats (Figure 6a). However, in the ObHIIT group this damage in obesity was reverted (ObHITT > Ob; *P* < 0.05), since the ObHIIT promoted an elevation of 23%. In addition, percentage shortening also exhibited a statistical difference between control groups, as the trained groups presented a higher percentage shortening than C (40%) (Figure 6a). When comparing only the trained groups, the CHIIT had a greater percentage shortening in relation to the ObHIIT group (21%). Regarding the maximum shortening and relaxation rates, the results demonstrate that the trained groups promoted an improvement in these parameters with an elevation of maximum shortening and relaxation rates compared to their respective sedentary groups (Figure 6b, c). There was no statistical difference between trained groups for these parameters (Figure 6d, e). Considering the time to 50% of peak shortening, it was observed that the Ob group presented a statistical reduction in this parameter when compared to C rats. Regarding the time to 50% of peak relaxation, the results showed that the ObHIIT group had a statistically longer time when compared to the CHIIT rats.

**FIGURE 7:**
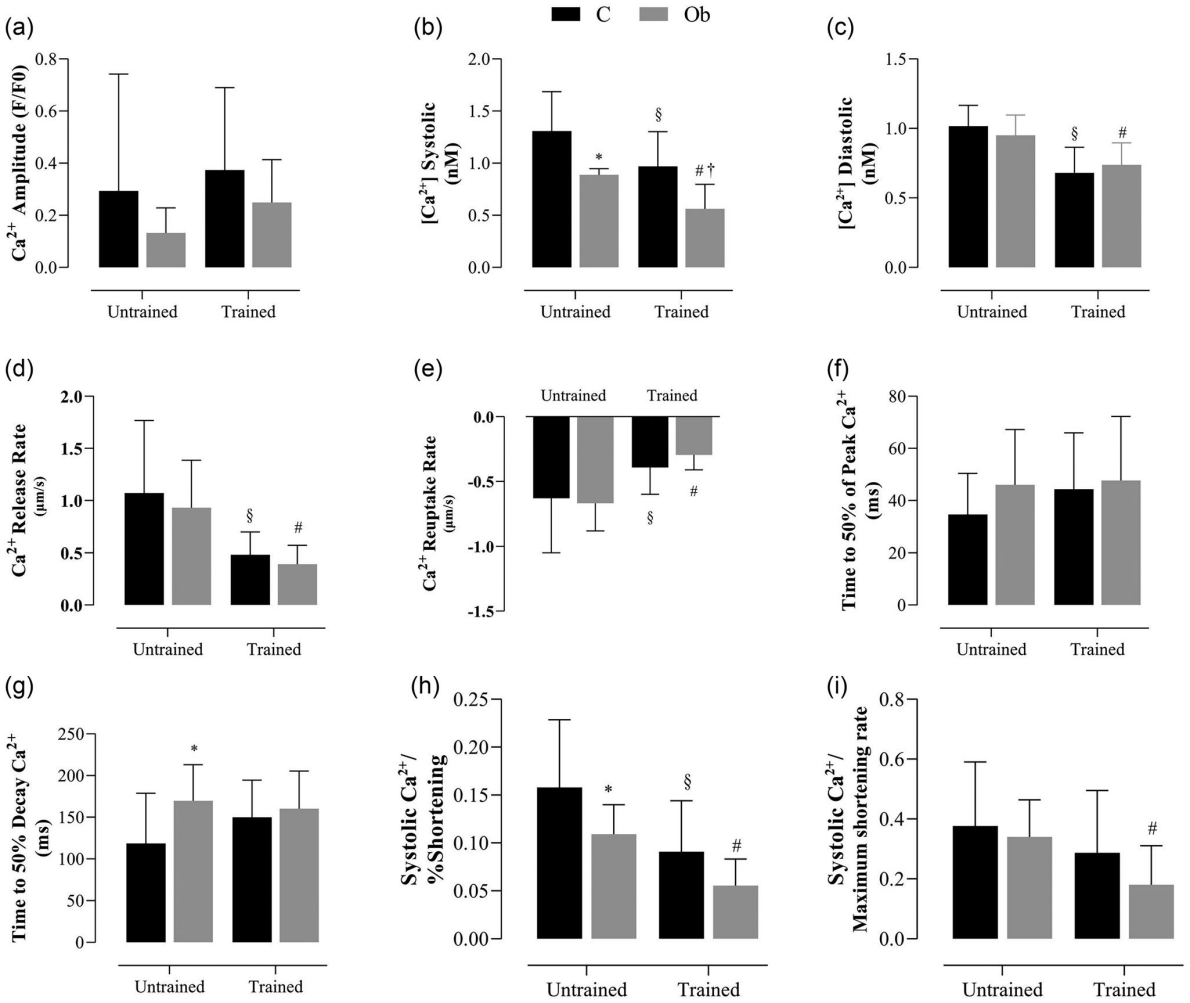
Effect of HIIT on intracellular Ca^2+^ handling of isolated cardiomyocytes stimulated at 1 Hz. (a) Calcium (Ca^2+^) transient amplitude. (b, c) Cardiomyocyte [Ca^2+^]_i_ systolic and diastolic. (d, e) Ca^2+^ release and reuptake rate. (f) Time to 50% peak Ca^2+^. (g) Time to 50% Ca^2+^ decay. (h, i) Evaluation of the responsiveness of myofilaments to Ca^2+^ assessed through the parameters of systolic Ca^2+^ to percentage shortening ratio (h) and systolic Ca^2+^/maximum shortening rate (i). Values expressed as means ± standard deviation. *P* < 0.05: *C versus Ob; ^§^C versus CHIIT; ^#^Ob versus ObHIIT; ^†^CHIIT versus ObHIIT. Two‐way ANOVA for independent samples, complemented with Tukey's *post‐hoc* test. C, sedentary control (*n* = 4, cells = 23); CHIIT, control submitted to high‐intensity interval training (*n* = 4, cells = 23); Ob, sedentary obese (*n* = 4, cells = 18); ObHIIT, obese submitted to high‐intensity interval training (*n* = 4, cells = 23).

Figure 7 illustrates the effects of HIIT on intracellular Ca^2+^ handling. Systolic Ca^2+^ levels (Figure 7b) were reduced in Ob and ObHIIT in relation to C and CHIIT groups, respectively. However, it was observed that the trained groups promoted a reduction in systolic Ca^2+^ when compared to their respective sedentary groups. Regarding the trained groups, it was observed that the CHIIT group had higher Ca^2+^ systolic levels when compared to the ObHIIT group. Additionally, C and Ob rats showed a greater increase in Ca^2+^ diastolic levels (Figure 7c) when compared to CHIIT and ObHIIT groups. No differences were found between trained groups for this parameter. Regarding the Ca^2+^ release and reuptake rates (Figure 7d, e), it was observed that C and Ob groups presented statistically higher rates when compared to CHIIT and ObHIIT groups, respectively. However, the results showed that the time to 50% Ca^2+^ decay (Figure 7g) was prolonged in the Ob group when compared to C. There were no alterations in Ca^2+^ amplitude (Figure 7a) and time to 50% of peak Ca^2+^ (Figure 7f) among groups.

Considering the systolic Ca^2+^ to percentage shortening ratio (Figure 7h), it was observed that group C presented statistically higher values when compared to Ob and CHIIT groups. Comparing the Ob and ObHIIT groups, HIIT was able to reduce the systolic Ca^2+^/percentage shortening ratio when compared to the Ob group. In relation to systolic Ca^2+^/maximum shortening rate (Figure 7i), a statistical difference was observed, as the ObHIIT group presented lower values in relation to the Ob group, indicating that HIIT enhanced the sensitivity of myofilaments to Ca^2+^ in obesity.

## 4 DISCUSSION

The purpose of the current study was to investigate the effects of HIIT on maximum oxygen consumption, a progressive physical performance test, nutritional, metabolic and hormonal profiles, as well as the cardiac remodelling process and intracellular Ca^2+^ handling in obese rats induced by a HFD rich in saturated fat.

The main findings of the present study indicate that HIIT, as a non‐pharmacological strategy for treating obesity, reduces visceral adipose area, enhances the cardiorespiratory condition (analysed by maximum ${\dot{V}}_{O_{2}}$, where Ob vs. ObHIIT is Δ60%), prevents the risk of IR, and increases the diameter of gastrocnemius fibres. Considering myocardial morphology and contractility, HIIT prevents LV interstitial fibrosis, improves cardiac contractility parameters (percentage shortening, maximum shortening and relaxation rates), and enhances myofilament sensitivity to Ca^2+^, as visualized by the efficiency in the release and recapture processes of cytosolic Ca^2+^ (reduced systolic Ca^2+^/percentage shortening and systolic Ca^2+^/maximum shortening rate). However, no significant changes in total body adiposity were observed. These findings suggest that HIIT promotes increased physical fitness, as well as positive changes in the cardiac remodelling process in obesity, preventing the incidence of metabolic changes and the risk of cardiovascular events. Additionally, in general, HIIT proved to be efficient in preventing cardiometabolic risks in the treated groups when compared to the sedentary groups.

Experimental models of obesity by HFD and/or high‐saturated fat/high‐sucrose diet have been extensively used in the literature to represent the aetiological profile of obesity developed in humans (Da Gamelin et al., 2016; Kelley et al. 2022; Silva et al., 2010). In this sense, we used a HFD rich in lard due to their high saturated fat content and obesogenic potential (Leopoldo et al., 2011). The high‐calorie diet employed in the current study effectively promoted obesity, as evidenced by a statistical difference in body weight observed in the third week of obesity induction; this difference was sustained until the completion of the obesity maintenance protocol. Consistent with previous investigations, HFDs that used lard as a fat source to develop the obesity were also able to increase body mass and adiposity (Dourmashkin et al., 2005; Lasker et al., 2019; Matias et al., 2018; Woods et al., 2003). In addition, Kelley et al. (2022) have also observed that rats fed a high‐saturated fat, high‐sucrose (HFHS) diet for ∼22 weeks had higher terminal body weights than rats fed a lean control diet, characterizing the obesity.

The treatment protocol involving the HIIT did not bring about positive changes in body adiposity in the Ob group, as indicated by the absence of alterations in body fat and the adiposity index, respectively. Nevertheless, a statistical difference in body weight and nutritional profile was observed only between the trained groups (ObHIIT > CHIIT), suggesting that the high‐calorie diet influenced this process. Factors contributing to the lack of effects of HIIT on adiposity are associated with the intrinsic physiological adaptations of the animals themselves. There was an increase in fat deposition and a potential lipogenic effect of insulin in the obese condition. Obesity led to an elevation in insulin levels (Figure 4), possibly contributing to greater fat accumulation and increased adiposity.

In light of this, the literature indicates that obesity is a condition capable of causing hyperglycaemia and hyperinsulinaemia (Ndumele et al., 2023). Elevated insulin levels, linked to the consumption of a HFD, result in the inhibition of the insulin receptor substrate/phosphoinositide 3‐kinase/Akt pathway. This pathway, responsible for activating insulin‐mediated glucose uptake, promotes the translocation of GLUT‐4 and GLUT‐2 to the membrane, facilitating glucose uptake. However, this pathway becomes inhibited by hyperinsulinaemia induced by obesity, leading to increased lipogenesis through the sterol regulatory element‐binding transcription factor 1 (SREBP1c). This process transforms circulating glucose into acetyl CoA through enzymatic esterification, subsequently forming fatty acids. These free fatty acids combine with glycerol, creating triacylglycerols stored in adipose tissue alongside other fatty acids, undergoing the same conjugation and formation process of triglycerides within the adipocyte. Consequently, there is an increase in body mass gain and adiposity (Zhai et al., 2023).

Differing from the nutritional profile data (Table 1), the histomorphological analysis revealed a statistical difference in the area and quantity of visceral fat adipocytes between the sedentary and trained obese groups. This observation indicates that HIIT was able to prevent adipocyte hypertrophy without reducing the overall number of adipocytes. Despite effectively preventing adipocyte hypertrophy, this non‐pharmacological intervention proved inefficient in reducing total body adiposity. The decrease in the visceral fat area can be attributed to the heightened sensitivity to catecholamines and the increased expression of β‐adrenergic receptors on the membrane of visceral fat adipocytes, rendering them more susceptible to lipolysis after HIIT sessions in adipose catabolism (Ndumele et al., 2023; Zhai et al., 2023).

Studies show that these effects persist even after HIIT sessions, since O_2_ consumption does not return to resting values immediately. This energy demand during the recovery period after exercise is known as excess post‐exercise oxygen consumption – EPOC. In this sense, it may exert a major impact on total energy expenditure, which favors the lipolysis and the reduction of visceral adipose tissue (Kemi et al., 2002, 2007; Wisløff et al., 2002, 2001). These findings suggest that HIIT was able to mobilize more visceral fat; however, it did not change total body adiposity, which was also composed of retroperitoneal and epididymal fat deposits.

A possible explanation for the lack of effective results in body adiposity after physical training with HIIT may be related to the reduction of β‐receptors in retroperitoneal and epididymal fat, which are less sensitive to the action of catecholamines (Bo et al., 2023; Yu et al., 2023). This information suggests and corroborates the findings of our study, since we did not find a decrease in total body adiposity, retroperitoneal and epididymal fat mass in the ObHIIT group; on the other hand, visceral fat mass presented lower values (Table 1). Therefore, these findings indicate the ineffectiveness of HIIT in reducing total body adiposity.

The assessment of maximum oxygen consumption and the progressive speed test has been utilized to measure physical fitness, becoming a widely used tool in studies involving both humans and animals. This analysis allows for the optimization of physical conditioning and the adjustment of training loads, thereby controlling intensity. It aids in the progression of speed during exercise sessions and physical performance tests (Wisløff et al., 2003). In the current study, HIIT promoted an increase in aerobic capacity in the trained groups compared to the untrained groups (${\dot{V}}_{O_{2}\max}$: ObHIIT vs. Ob, Δ 60%) and (${\dot{V}}_{O_{2}\max}$: CHIIT vs. C, Δ 43.5%), exhibiting improved values in ${\dot{V}}_{O_{2}}$ baseline, speed, distance and time in relation to the sedentary groups. Therefore, our findings are in fact the result of physical training using HIIT as a non‐pharmacological strategy, which can be effective for improvement of cardiorespiratory health in obesity. Mendes et al. (2022) demonstrated that among the trained groups, there was a reduction in body mass and the size of the adipocyte area, suggesting that interval sessions contribute to improved cardiometabolic health, particularly in preventing obesity and IR.

Corroborating our findings, França et al. (2020) and Khalafi et al. (2020) have also observed that HIIT was effective in improving parameters related to physical performance in obese rats induced by HFD. In this study, it was found that HIIT promoted better physical performance with an increase in speed, distance and time parameters, as well as higher ${\dot{V}}_{O_{2}\max}$ values in HIIT obese animals. Nevertheless, Costa et al. (2021), using HIIT obese rats, showed an improvement of maximum exercise capacity, but there was no difference in relation untrained obese rats the post‐exercise condition. Delpisheh and Safarzade (2022) showed that obese rats fed a high‐fat, high‐sucrose diet also presented an elevation in aerobic capacity after performing 8 weeks of HIIT. Another important aspect that we demonstrated in the current study for maximum oxygen consumption was that the same pattern was observed between the trained groups (higher ${\dot{V}}_{O_{2}\max}$) and the sedentary groups (modest reduction in ${\dot{V}}_{O_{2}\max}$).

Another effect observed with HIIT was the increase in muscle mass, evidenced by the greater muscle fibre diameter in the trained groups compared to the sedentary groups. In this context, HIIT was effective in inducing skeletal muscle hypertrophy. Despite not finding a statistical difference, our study revealed higher mass values for the gastrocnemius muscle in the trained groups compared to the sedentary groups. However, in the histomorphological analysis, the gastrocnemius fibre diameter was greater in the trained groups compared to the sedentary groups.

The GTT is a laboratory examination used to check for disturbances in glucose metabolism and tendencies toward diabetes mellitus. Additionally, the ITT is a tool employed to assess the increase in insulin in response to glucose overload. Analysing this information together, the results of the present study suggest that HIIT was not effective in attenuating glycaemic values in our obesity model after the intraperitoneal GTT and ITT tests. In contrast to our findings, Mendes et al. (2022) observed that HIIT reduced fasting glucose levels, decreased glucose intolerance and improved insulin tolerance. The authors suggest that one possible mechanism by which HIIT promotes this effect is by increasing the expression and translocation of GLUT‐2 and GLUT‐4, as well as improving IR. The physiological mechanisms responsible for improvements in glycaemic control and insulin sensitivity after a period of the HIIT protocol are not fully elucidated in the literature.

Although we did not observe statistical differences between the glycaemic curve values and insulin increase in response to glucose overload, our study showed a statistical difference in plasma insulin values and HOMA‐IR and HOMA‐β parameters. The findings demonstrate that the trained groups presented the lowest values of the parameters mentioned above, suggesting a protective effect on IR; in this sense, HIIT was effective in improving insulin sensitivity, promoting lower values of basal insulin, HOMA‐IR and HOMA‐β, corroborating the findings of ${\dot{V}}_{O_{2}\max}$. In line with our findings, Ghiasi et al. (2015), investigated the effects of physical training on the glucose profile of Ob rats induced by a HFD and found that physical training significantly improved the parameters of HOMA‐IR, oral glucose tolerance test (OGTT), HbA1C and basal/fasting insulin compared to those who did not undergo the same treatment. The authors conclude that HIIT is an important tool for managing hyperglycaemia and diabetes mellitus conditions.

Lipid profile and cardiac injury markers are used as tools to verify disturbances in lipid metabolism and damage to cardiac muscle cells. In the current study, the effects of HIIT on these lipid parameters were not observed. However, when comparing the trained groups, it was noted that the CHIIT group had higher HDL‐c rates compared to the ObHIIT group, suggesting that obesity is a preponderant factor in the reduction of this lipoprotein. In contrast to our findings, in the study of Bo et al. (2023), which investigated the effect of HIIT on the adiposity and lipid profile of obese Wistar rats, it was observed that the consumption of a HFD is associated with the development of dyslipidaemia. The authors showed that sedentary obese animals had statistically higher levels of cholesterol and serum triacylglycerols, and it has been observed that HIIT is an essential non‐pharmacological tool for the management of dyslipidaemia.

Cardiac remodelling is defined as a set of morphological, functional, molecular, cellular and interstitial cardiac changes that are clinically manifested by alterations in the mass, size and function of the heart in response to physiological and/or pathological aggression (Abel et al., 2008). Regarding data on heart mass and cardiac injury markers (CK‐MB), our results demonstrate that the HIIT protocol was not able to modify macroscopic parameters and did not promote alterations in cardiac damage markers at obesity (Ob vs. ObHIIT; *P* = 0.09). However, in the histomorphological analysis, the trained groups exhibited cardiac hypertrophy, as observed by the higher CSA when compared to the sedentary groups, suggesting a physiological adaptation. Additionally, this non‐pharmacological tool reduced the interstitial collagen fraction, preventing cardiac fibrosis in obesity. These findings highlight the physiological effect of HIIT on the cardiovascular adaptation process. Corroborating our findings, Engel et al. (2022), based on morpho‐anatomical analyses and conventional thoracic echocardiography of obese rats submitted to an 8‐week HIIT training programme, found that the HIIT protocol promotes cardiac hypertrophy with a consequent improvement in functional capacity. These results align with our findings and the existing literature, which demonstrates induced ventricular hypertrophy in rodents (Kemi et al., 2002, 2007; Wisløff et al., 2002, 2001, 2003).

Regarding the cardiac function in obesity, most studies found myocardial dysfunction from different types of hypercaloric diets and preparations involving the heart (Axelsen et al., 2010; Ferron et al., 2015; França et al., 2020; Gregolin et al., 2024; Relling et al., 2006). Boldt et al. (2021) showed that the cardiac muscle from animals exposed to an obesogenic diet for 24 weeks had impaired contractile properties, which were partially prevented by a 12‐week aerobic exercise regime. Another study conducted by Gregolin et al. (2024) demonstrated that sucrose induced cardiac dysfunction and decreased myocardial contractility in obese rats. Nevertheless, a previous study from our laboratory did not observe cardiac dysfunction or impaired intracellular Ca^2+^ handling proteins in obese rats (de Deus et al., 2019).

Several studies have demonstrated that cardiac contractility is directly related to intracellular Ca^2+^ homeostasis (Abel et al., 2008; Guatimosim et al., 2001; Leopoldo et al., 2011; Wisløff et al., 2003). However, few studies have investigated the effects of HIIT or others exercise approaches on obesity‐induced contractile dysfunction, specifically addressing Ca^2+^ handling (Boldt et al., 2021; Gregolin et al., 2024). In this context, the current study showed that HIIT increased the percentage shortening (CHIIT vs. C, 40%; ObHIIT vs. Ob, 23%), as well as maximum shortening and relaxation rates, suggesting a pronounced effect of HIIT on myocardial function. Wisloff et al. (2003) suggested that the greater cell shortening can be related to higher sensitivity to Ca^2+^, as well as the induction of adaptive hypertrophy in cardiac myocytes with improved contractile function.

Considering the intracellular Ca^2+^ handling, HIIT also resulted in a reduction in diastolic and systolic Ca^2+^ concentrations, but with a decrease in the release and reuptake rates of intracellular Ca^2+^. Nevertheless, we also observed a reduction in the systolic Ca^2+^/percentage shortening and systolic Ca^2+^/maximum shortening rate ratio when compared to the Ob group. These findings demonstrate that HIIT was effective in promoting enhanced sensitivity of myofilaments to Ca^2+^ in obesity. In this context, we can say that the greater cardiomyocyte contractility in ObHIIT rats was associated to lower systolic Ca^2+^ concentration, possibly due to higher sensitivity to Ca^2+^. These results are in accordance with several studies that have consistently shown that exercise increases the Ca^2+^ sensitivity of cardiac muscle (Diffee & Nagle, 2003; Diffee et al., 2001). However, Boldt el al. (2021), using high fat, high sucrose rats subjected to running exercise training for 12 weeks, did not show elevation of Ca^2+^ sensitivity. The authors suggested that the absence of this adaptation in these animals may be an indication that obesity inhibited the expression of the positive effects of exercise on Ca^2+^ sensitivity in their study.

Also, in agreement to our findings, Kemi et al. (2007) investigated whether CaMK‐mediated changes in Ca^2+^ signalling contribute to the effects mediated by physical exercise on inotropy and lusitropy in cardiomyocytes from Wistar rats. These authors have found that healthy animals undergoing HIIT training showed greater percentage contractility and faster relaxation rates but did not increase maximum systolic and diastolic Ca^2+^ levels; these changes were also associated with higher sensitivity of intracellular Ca^2+^ myofilament. Our findings regarding the maximum shortening and relaxation rates and the intracellular Ca^2+^ handling showed that the ObHIIT group presented lower shortening and relaxation rates, without a prolongation of times to 50% of peak and decay in Ca^2+^.

Increased values of systolic and diastolic Ca^2+^, as well as the release rate, are associated with a greater risk of cardiomyocyte apoptosis and cellular toxicity due to the greater influx of calcium into the nucleus, resulting in cell death. These conditions of increased intracellular Ca^2+^ influx suggest possible dysfunction in cellular relaxation, which may be related to the reduced activity of sarcoplasmic reticulum Ca^2+^‐ATPase (SERCA), phospholamban (PLB), or Na^+^/Ca^2+^ exchanger 2 (NCX). Therefore, the use of the HIIT protocol in experimental models of obesity has been developed and described in the literature with the aim of elucidating the improvement of intracellular Ca^2+^ handling and its molecular mechanisms in the context of obesity (Abel et al., 2008; Kemi et al., 2007; Kemi, 2023; Leopoldo et al., 2011).

It is worth noting that the reduction in Ca^2+^ release and reuptake rates in the current study is related to the decrease in systolic and diastolic Ca^2+^ levels without alterations in times to 50% peak and decay Ca^2+^, which may suggest better sensitivity to Ca^2+^ due to a greater expression and/or activity of L‐type Ca^2+^ channels and regulatory proteins of Ca^2+^ handling. However, it was not possible to analyse the expression of proteins regulating intracellular Ca^2+^ handling in this study.

In conclusion, HIIT was efficient in cardiomyocyte contractility, promoting physiological cardiac remodelling with improved contractile functional parameters and enhanced sensitivity of myofilaments to Ca^2+^ in obesity. Additionally, HIIT demonstrated improvements in cardiorespiratory parameters and physical performance, reduced visceral fat area, and prevented the risk of IR and LV interstitial fibrosis. However, HIIT was not able to reduce total body adiposity.

## AUTHOR CONTRIBUTIONS

Matheus Corteletti dos Santos, Daniel Sesana da Silva, Jóctan Pimentel Cordeiro, Danilo Sales Bocalini, Ana Paula Lima Leopoldo, and André Soares Leopoldo conceived the design of the study. Matheus Corteletti dos Santos, Daniel Sesana da Silva, Jóctan Pimentel Cordeiro, Ezio Henrique da Silva Gomes, Lucas Furtado Domingos, and Breno Valentim Nogueira performed the experiments. Matheus Corteletti dos Santos, Breno Valentim Nogueira, Danilo Sales Bocalini, Ana Paula Lima Leopoldo, and André Soares Leopoldo interpreted, discussed the data, wrote the manuscript and statistical analysis. All authors revised the manuscript and the final version of the manuscript. All authors have read and approved the final version of this manuscript and agree to be accountable for all aspects of the work in ensuring that questions related to the accuracy or integrity of any part of the work are appropriately investigated and resolved. All persons designated as authors qualify for authorship, and all those who qualify for authorship are listed.

## CONFLICT OF INTEREST

The authors declare that there was no conflict of interest.

## Data Availability

The data sets used and/or data analysed during the current study are available from the corresponding author upon reasonable request.
